# 3-[(2-Formyl­thio­phen-3-yl)(hy­droxy)meth­yl]thio­phene-2-carbaldehyde

**DOI:** 10.1107/S1600536811052500

**Published:** 2011-12-21

**Authors:** Lili Sun, Guijuan Li, Qing Su

**Affiliations:** a School of Chemical Engineering, Changchun University of Technology, Changchun 130012, People’s Republic of China; b School of Chemistry, Jilin University, Changchun 130012, People’s Republic of China

## Abstract

In the title compound, C_11_H_8_O_3_S_2_, the dihedral angle between the mean planes of the two thio­phene rings is 65.10 (10)°. Intra­molecular C—H⋯O inter­actions form *S*(6) and *S*(7) ring motifs. In the crystal, chains along the *a* axis are formed by C—H⋯O inter­actions. Adjacent chains are connected into a three-dimensional network by C—H⋯O and O—H⋯O inter­actions.

## Related literature

For details and applications of thio­phene-based aldehydes, see: Basu & Das (2011 ▶); Guarín *et al.* (2007 ▶); Herbivo *et al.* (2009 ▶); Jain *et al.* (2010 ▶). For hydrogen-bonding motifs, see: Bernstein *et al.* (1995 ▶). For optical applications of formyl thio­phene derivatives, see: Raposo & Kirsch (2003 ▶); Raposo *et al.* (2004 ▶). For related Schiff base compounds reported by our group, see: Su *et al.* (2007*a* ▶,**b* ▶,c* ▶, 2009 ▶).
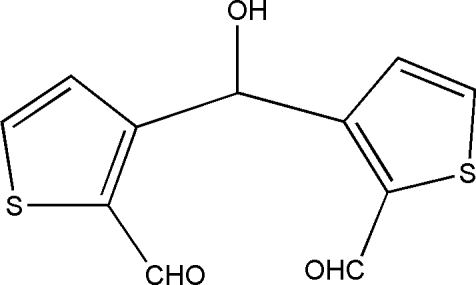


## Experimental

### 

#### Crystal data

C_11_H_8_O_3_S_2_
*M* *_r_* = 252.29
Monoclinic, 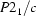

*a* = 7.6227 (18) Å
*b* = 10.136 (2) Å
*c* = 14.272 (3) Å
β = 101.998 (4)°
*V* = 1078.7 (4) Å^3^
*Z* = 4
Mo *K*α radiation
μ = 0.48 mm^−1^
*T* = 293 K
0.26 × 0.24 × 0.21 mm

#### Data collection

Bruker SMART APEX CCD area-detector diffractometer
Absorption correction: multi-scan (*SADABS*; Bruker, 2001 ▶) *T*_min_ = 0.886, *T*_max_ = 0.906
6687 measured reflections
2122 independent reflections
1798 reflections with *I* > 2σ(*I*)
*R*_int_ = 0.025

#### Refinement

*R*[*F* ^2^ > 2σ(*F* ^2^)] = 0.041
*wR*(*F* ^2^) = 0.100
*S* = 1.03
2122 reflections
145 parameters
H-atom parameters constrained
Δρ_max_ = 0.31 e Å^−3^
Δρ_min_ = −0.17 e Å^−3^

### 

Data collection: *SMART* (Bruker, 1998 ▶); cell refinement: *SAINT* (Bruker, 1998 ▶); data reduction: *SAINT*; program(s) used to solve structure: *SHELXS97* (Sheldrick, 2008 ▶); program(s) used to refine structure: *SHELXL97* (Sheldrick, 2008 ▶); molecular graphics: *SHELXTL* (Sheldrick, 2008 ▶); software used to prepare material for publication: *SHELXTL*.

## Supplementary Material

Crystal structure: contains datablock(s) global, I. DOI: 10.1107/S1600536811052500/mw2040sup1.cif

Structure factors: contains datablock(s) I. DOI: 10.1107/S1600536811052500/mw2040Isup2.hkl

Supplementary material file. DOI: 10.1107/S1600536811052500/mw2040Isup3.cml

Additional supplementary materials:  crystallographic information; 3D view; checkCIF report

## Figures and Tables

**Table 1:** Hydrogen-bond geometry (Å, °)

*D*—H⋯*A*	*D*—H	H⋯*A*	*D*⋯*A*	*D*—H⋯*A*
C6—H6⋯O2	0.98	2.43	3.101 (3)	125
C11—H11⋯O2	0.93	2.50	3.261 (3)	139
C4—H4⋯O1^i^	0.93	2.55	3.377 (3)	149
C9—H9⋯O2^ii^	0.93	2.57	3.458 (3)	160
C10—H10⋯O3^ii^	0.93	2.55	3.397 (3)	152
O1—H1⋯O3^iii^	0.82	2.03	2.825 (2)	162

Symmetry codes: (i) 
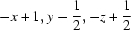
; (ii) 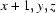; (iii) 
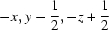
.

**Fig. 1.**
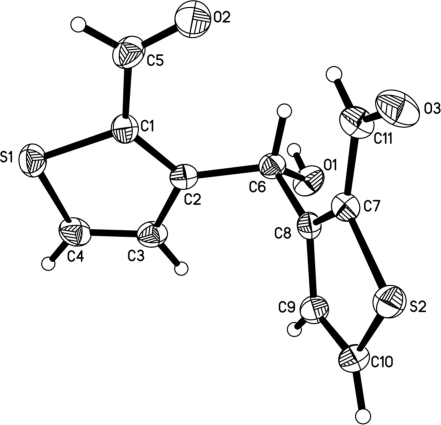
View of the molecule of (I) showing the atom labelling scheme. Displacement ellipsoids are drawn at the 30% probability level.

**Fig. 2.**
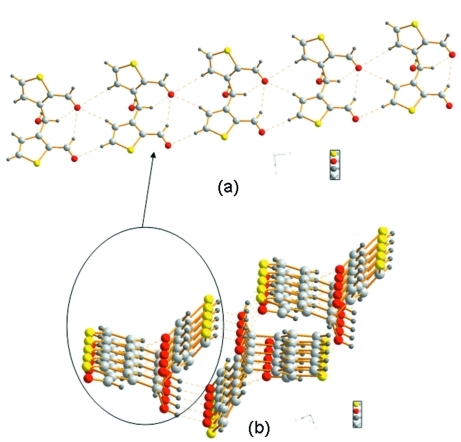
The molecular packing of (I). (a) shows the 1-D chain formed by intramolecular and intermolecular C—H···O interactions. (b) shows the 3-D network formed by 1-D chains through further intermolecular C4—H4···O1 and O1—H1···O3 interactions. Hydrogen bonds are indicated by dashed lines.

## supplementary crystallographic information

### Comment

Thiophene aldehydes and their homologues are an important class of organic compounds. Some of them can be used as precursors for syntheses of azomethines (also named Schiff) (Guarín *et al.*, 2007; Basu *et al.*, 2011) thiacarbaporphyrins (Jain *et al.*, 2010), and dicyanovinyl-derivatives (Raposo *et al.*, 2003, 2004) for optical applications. We are interested in the structures and properties of Schiff base ligands and their metal complexes (Su, Wu, Li, *et al.* 2007*a*, 2007*b*; Su, Gao, *et al.* 2007*c*; Su *et al.* 2009). Herein, a new thiophene dialdehyde, of which the aldehyde group can easily react with all kinds of arylamines to form Schiff-bases, was synthesized and the crystal structure of the title compound, (I) (Fig. 1), is reported.

In the title molecule, Fig. 1, the angle between the mean planes of the two thiophene rings is 65.1°. The two aldehyde groups are nearly coplanar with the thiophene rings to which they are attached. The C3–C2–C1–C5, C6–C2–C3–C4, C6–C8–C9–C10 and C9–C8–C7–C11 torsion angles are -174.3 (2), -178.46 (19), 178.91 (18) and 178.7 (2)°, respectively. Both S(6) and S(7) ring motifs (Bernstein *et al.*, 1995) are formed due to intramolecular C—H···O interactions (Fig. 2 and Table 1). In the crystal there exist intermolecular C—H···O interactions with the graph-set motifs  *R*_2_^1^(8) and *R*_2_^2^(13) (Bernstein *et al.*, 1995) which form one-dimensional chains along the *a* axis (Fig. 2a and Table 1). The adjacent chains are connected into a 3-dimensional network by intermolecular C4—H4···O1 and O1—H1···O3 interactions (Fig. 2b and Table 1).

### Experimental

Compound (I) was synthesized from thiophene-3-carbaldehyde, *n*-BuLi, 3-bromothiophene and ethyl formate *via* a one-pot reaction (manuscript in preparation). It was crystallized slowly from ethanol at 298 K.

### Refinement

The C-bound H atoms were positioned geometrically with C—H = 0.93 (aromatic and carbonyl carbons) and 0.98 (methine) Å, and allowed to ride on their parent atoms in the riding model approximation with *U*_iso_(H) = 1.2 *U*_eq_(C). The atom H1 was located in a difference map and included as a riding contribution with O—H adjusted to 0.82 Å and with *U*U_iso_(H) = 1.2 *U*U_eq_(O).

### Crystal data

**Table:**

C_11_H_8_O_3_S_2_	*F*(000) = 520
*M**_r_* = 252.29	*D*_x_ = 1.554 Mg m^−^^3^
Monoclinic, *P*2_1_/*c*	Mo *K*α radiation, λ = 0.71073 Å
Hall symbol: -P 2ybc	Cell parameters from 2097 reflections
*a* = 7.6227 (18) Å	θ = 2.5–26.1°
*b* = 10.136 (2) Å	µ = 0.48 mm^−^^1^
*c* = 14.272 (3) Å	*T* = 293 K
β = 101.998 (4)°	Block, yellow
*V* = 1078.7 (4) Å^3^	0.26 × 0.24 × 0.21 mm
*Z* = 4	

### Data collection

**Table:**

Bruker SMART APEX CCD area-detector diffractometer	2122 independent reflections
Radiation source: fine-focus sealed tube	1798 reflections with *I* > 2σ(*I*)
graphite	*R*_int_ = 0.025
Detector resolution: 9.00 pixels mm^-1^	θ_max_ = 26.1°, θ_min_ = 2.5°
phi and ω scans	*h* = −8→9
Absorption correction: multi-scan (*SADABS*; Bruker, 2001)	*k* = −10→12
*T*_min_ = 0.886, *T*_max_ = 0.906	*l* = −17→17
6687 measured reflections	

### Refinement

**Table:**

Refinement on *F*^2^	Primary atom site location: structure-invariant direct methods
Least-squares matrix: full	Secondary atom site location: difference Fourier map
*R*[*F*^2^ > 2σ(*F*^2^)] = 0.041	Hydrogen site location: inferred from neighbouring sites
*wR*(*F*^2^) = 0.100	H-atom parameters constrained
*S* = 1.03	*w* = 1/[σ^2^(*F*_o_^2^) + (0.0511*P*)^2^ + 0.3301*P*] where *P* = (*F*_o_^2^ + 2*F*_c_^2^)/3
2122 reflections	(Δ/σ)_max_ < 0.001
145 parameters	Δρ_max_ = 0.31 e Å^−^^3^
0 restraints	Δρ_min_ = −0.17 e Å^−^^3^

### Special details

**Table:**

Geometry. All esds (except the esd in the dihedral angle between two l.s. planes) are estimated using the full covariance matrix. The cell esds are taken into account individually in the estimation of esds in distances, angles and torsion angles; correlations between esds in cell parameters are only used when they are defined by crystal symmetry. An approximate (isotropic) treatment of cell esds is used for estimating esds involving l.s. planes.
Refinement. Refinement of F^2^ against ALL reflections. The weighted R-factor wR and goodness of fit S are based on F^2^, conventional R-factors R are based on F, with F set to zero for negative F^2^. The threshold expression of F^2^ > 2sigma(F^2^) is used only for calculating R-factors(gt) etc. and is not relevant to the choice of reflections for refinement. R-factors based on F^2^ are statistically about twice as large as those based on F, and R- factors based on ALL data will be even larger.

### Fractional atomic coordinates and isotropic or equivalent isotropic displacement parameters (Å^2^)

**Table:**

	*x*	*y*	*z*	*U*_iso_*/*U*_eq_	
S1	0.18693 (8)	0.05474 (6)	0.40611 (4)	0.0483 (2)	
S2	0.35779 (8)	0.71529 (6)	0.45073 (4)	0.04438 (19)	
O1	0.2719 (2)	0.41275 (16)	0.18884 (10)	0.0461 (4)	
H1	0.2189	0.3605	0.1490	0.069*	
O3	−0.0407 (2)	0.72325 (18)	0.41728 (12)	0.0549 (5)	
C2	0.2269 (3)	0.2727 (2)	0.32022 (14)	0.0333 (5)	
O2	−0.1559 (2)	0.3386 (2)	0.33753 (15)	0.0696 (6)	
C1	0.1038 (3)	0.2063 (2)	0.36192 (15)	0.0364 (5)	
C8	0.2905 (3)	0.5135 (2)	0.33862 (14)	0.0328 (5)	
C11	0.0174 (3)	0.6327 (2)	0.37609 (15)	0.0434 (5)	
H11	−0.0644	0.5759	0.3388	0.052*	
C7	0.2056 (3)	0.6090 (2)	0.38216 (14)	0.0356 (5)	
C5	−0.0793 (3)	0.2385 (3)	0.36692 (18)	0.0480 (6)	
H5	−0.1425	0.1769	0.3951	0.058*	
C9	0.4790 (3)	0.5276 (2)	0.36272 (15)	0.0391 (5)	
H9	0.5582	0.4716	0.3407	0.047*	
C4	0.3810 (3)	0.0795 (2)	0.36802 (17)	0.0487 (6)	
H4	0.4740	0.0184	0.3759	0.058*	
C6	0.1976 (3)	0.4072 (2)	0.27314 (14)	0.0344 (5)	
H6	0.0688	0.4260	0.2560	0.041*	
C10	0.5322 (3)	0.6320 (2)	0.42159 (16)	0.0435 (5)	
H10	0.6517	0.6553	0.4436	0.052*	
C3	0.3852 (3)	0.1982 (2)	0.32482 (16)	0.0424 (5)	
H3	0.4825	0.2276	0.3006	0.051*	

### Atomic displacement parameters (Å^2^)

**Table:**

	*U*^11^	*U*^22^	*U*^33^	*U*^12^	*U*^13^	*U*^23^
S1	0.0545 (4)	0.0366 (4)	0.0535 (4)	0.0026 (3)	0.0109 (3)	0.0055 (3)
S2	0.0443 (3)	0.0373 (3)	0.0493 (4)	−0.0010 (2)	0.0045 (2)	−0.0050 (3)
O1	0.0513 (10)	0.0534 (10)	0.0367 (8)	−0.0124 (8)	0.0166 (7)	−0.0050 (7)
O3	0.0456 (10)	0.0604 (12)	0.0575 (10)	0.0198 (8)	0.0077 (8)	−0.0096 (9)
C2	0.0316 (11)	0.0347 (12)	0.0339 (11)	−0.0004 (8)	0.0072 (8)	−0.0061 (9)
O2	0.0489 (11)	0.0614 (13)	0.1079 (16)	0.0157 (9)	0.0376 (10)	0.0192 (11)
C1	0.0396 (11)	0.0329 (12)	0.0374 (11)	0.0024 (9)	0.0094 (9)	−0.0012 (9)
C8	0.0331 (11)	0.0305 (11)	0.0348 (10)	0.0013 (8)	0.0073 (8)	0.0064 (8)
C11	0.0395 (12)	0.0456 (14)	0.0435 (12)	0.0073 (10)	0.0050 (10)	−0.0002 (10)
C7	0.0346 (11)	0.0351 (12)	0.0355 (11)	0.0012 (9)	0.0040 (8)	0.0025 (9)
C5	0.0430 (13)	0.0479 (15)	0.0586 (15)	−0.0019 (11)	0.0229 (11)	0.0016 (12)
C9	0.0322 (11)	0.0417 (13)	0.0451 (12)	0.0005 (9)	0.0118 (9)	0.0047 (10)
C4	0.0418 (13)	0.0418 (14)	0.0599 (15)	0.0112 (10)	0.0047 (11)	−0.0057 (11)
C6	0.0306 (10)	0.0379 (12)	0.0358 (11)	−0.0001 (9)	0.0094 (8)	0.0012 (9)
C10	0.0326 (11)	0.0455 (14)	0.0513 (13)	−0.0065 (10)	0.0061 (9)	0.0060 (11)
C3	0.0327 (11)	0.0430 (13)	0.0519 (13)	0.0018 (10)	0.0098 (10)	−0.0056 (11)

### Geometric parameters (Å, °)

**Table:**

S1—C4	1.698 (3)	C8—C9	1.414 (3)
S1—C1	1.730 (2)	C8—C6	1.503 (3)
S2—C10	1.698 (2)	C11—C7	1.439 (3)
S2—C7	1.730 (2)	C11—H11	0.9300
O1—C6	1.434 (2)	C5—H5	0.9300
O1—H1	0.8200	C9—C10	1.359 (3)
O3—C11	1.222 (3)	C9—H9	0.9300
C2—C1	1.386 (3)	C4—C3	1.355 (3)
C2—C3	1.414 (3)	C4—H4	0.9300
C2—C6	1.515 (3)	C6—H6	0.9800
O2—C5	1.201 (3)	C10—H10	0.9300
C1—C5	1.450 (3)	C3—H3	0.9300
C8—C7	1.383 (3)		
			
C4—S1—C1	91.70 (11)	C1—C5—H5	117.4
C10—S2—C7	91.10 (11)	C10—C9—C8	112.8 (2)
C6—O1—H1	109.5	C10—C9—H9	123.6
C1—C2—C3	111.6 (2)	C8—C9—H9	123.6
C1—C2—C6	125.23 (18)	C3—C4—S1	112.42 (18)
C3—C2—C6	123.17 (19)	C3—C4—H4	123.8
C2—C1—C5	131.0 (2)	S1—C4—H4	123.8
C2—C1—S1	111.02 (16)	O1—C6—C8	106.03 (16)
C5—C1—S1	117.75 (17)	O1—C6—C2	111.10 (17)
C7—C8—C9	111.52 (19)	C8—C6—C2	111.21 (16)
C7—C8—C6	125.27 (18)	O1—C6—H6	109.5
C9—C8—C6	123.21 (19)	C8—C6—H6	109.5
O3—C11—C7	123.5 (2)	C2—C6—H6	109.5
O3—C11—H11	118.2	C9—C10—S2	112.97 (17)
C7—C11—H11	118.2	C9—C10—H10	123.5
C8—C7—C11	130.1 (2)	S2—C10—H10	123.5
C8—C7—S2	111.65 (15)	C4—C3—C2	113.3 (2)
C11—C7—S2	118.27 (17)	C4—C3—H3	123.4
O2—C5—C1	125.2 (2)	C2—C3—H3	123.4
O2—C5—H5	117.4		
			
C3—C2—C1—C5	−174.3 (2)	C7—C8—C9—C10	−0.8 (3)
C6—C2—C1—C5	4.5 (4)	C6—C8—C9—C10	178.91 (18)
C3—C2—C1—S1	0.4 (2)	C1—S1—C4—C3	0.90 (19)
C6—C2—C1—S1	179.10 (16)	C7—C8—C6—O1	129.0 (2)
C4—S1—C1—C2	−0.71 (16)	C9—C8—C6—O1	−50.6 (2)
C4—S1—C1—C5	174.72 (19)	C7—C8—C6—C2	−110.1 (2)
C9—C8—C7—C11	178.7 (2)	C9—C8—C6—C2	70.2 (2)
C6—C8—C7—C11	−1.0 (4)	C1—C2—C6—O1	−141.08 (19)
C9—C8—C7—S2	0.6 (2)	C3—C2—C6—O1	37.5 (3)
C6—C8—C7—S2	−179.07 (15)	C1—C2—C6—C8	101.1 (2)
O3—C11—C7—C8	−178.5 (2)	C3—C2—C6—C8	−80.3 (2)
O3—C11—C7—S2	−0.5 (3)	C8—C9—C10—S2	0.6 (3)
C10—S2—C7—C8	−0.24 (17)	C7—S2—C10—C9	−0.21 (18)
C10—S2—C7—C11	−178.60 (18)	S1—C4—C3—C2	−0.9 (3)
C2—C1—C5—O2	−4.2 (4)	C1—C2—C3—C4	0.3 (3)
S1—C1—C5—O2	−178.5 (2)	C6—C2—C3—C4	−178.46 (19)

### Hydrogen-bond geometry (Å, °)

**Table:**

*D*—H···*A*	*D*—H	H···*A*	*D*···*A*	*D*—H···*A*
C6—H6···O2	0.98	2.43	3.101 (3)	125
C11—H11···O2	0.93	2.50	3.261 (3)	139
C4—H4···O1^i^	0.93	2.55	3.377 (3)	149
C9—H9···O2^ii^	0.93	2.57	3.458 (3)	160
C10—H10···O3^ii^	0.93	2.55	3.397 (3)	152
O1—H1···O3^iii^	0.82	2.03	2.825 (2)	162

Symmetry codes: (i) −*x*+1, *y*−1/2, −*z*+1/2; (ii) *x*+1, *y*, *z*; (iii) −*x*, *y*−1/2, −*z*+1/2.
